# A Recurrent Cryptic *MED14-HOXA9* Rearrangement in an Adult Patient With Mixed-Phenotype Acute Leukemia, T/myeloid, NOS

**DOI:** 10.3389/fonc.2021.690218

**Published:** 2021-07-22

**Authors:** Qian Wang, Ling Zhang, Ming-qing Zhu, Zhao Zeng, Bao-zhi Fang, Jun-dan Xie, Jin-lan Pan, Chun-xiao Wu, Ni Wu, Ri Zhang, Su-ning Chen, Hai-ping Dai

**Affiliations:** ^1^National Clinical Research Center for Hematologic Diseases, Jiangsu Institute of Hematology, The First Affiliated Hospital of Soochow University, Suzhou, China; ^2^Department of Hematology, The Affiliated Suzhou Hospital of Nanjing Medical University (Main part of Suzhou Municipal Hospital), Suzhou, China; ^3^Institute of Blood and Marrow Transplantation, Collaborative Innovation Center of Hematology, Soochow University, Suzhou, China

**Keywords:** mixed-phenotype acute leukemia, *MED14-HOXA9*, fusion gene, *PTPN11*, *NOTCH1*

## Abstract

To define the fusion genes in T/myeloid mixed-phenotype acute leukemia (T/M MPAL), we performed transcriptome sequencing of diagnostic bone marrow samples from 20 adult patients. Our analysis identified a second instance of a recurrent *MED14-HOXA9* chimeric gene resulting from the in-frame fusion of exon 23 of *MED14* and exon 1 of *HOXA9*, the first in an adult patient. The *MED14-HOXA9* fusion gene was detected in both the diagnostic and relapsed blasts with reverse transcription-polymerase chain reaction and Sanger sequencing. The patient received combined conventional chemotherapy but suffered relapse at 11 months and died of disease progression one year after the initial diagnosis. Our data suggest that *MED14-HOXA9* is a cryptic recurrent aberration in T/M MPAL, which might indicate an aggressive clinical course and inferior outcome after conventional chemotherapy. Further studies will be carried out to reveal the effects of the *MED14-HOXA9* fusion on the differentiation and proliferation of leukemia stem cells, as well as suitable treatment strategies for this emerging entity.

## Introduction

T/myeloid mixed-phenotype acute leukemia (T/M MPAL) is a rare malignancy responsible for approximately 1% of all leukemia cases and is characterized by leukemic blasts presenting both T lineage and myeloid markers (1). Only a few patients with T/M MPAL have been identified with recurrent genetic aberrations, such as the t(9;22)(q34.1;q11.2)/*BCR-ABL1* and t(v;11q23.3)/*KMT2A* rearrangements (2). Most T/M MPAL cases carry nonspecific clonal chromosomal abnormalities, lack uniform treatment strategies and carry unfavorable prognoses (3, 4). Using next-generation and transcriptome sequencing, Takahashi and Alexander et al. elucidated the genetic and epigenetic heterogeneity of MPAL in a case series (5, 6), but only a few of the patients had T/M MPAL. For a better understanding of the genomic landscape of T/M MPAL, we performed transcriptome sequencing of diagnostic blasts from 20 adult patients with normal karyotype or karyotype failure and, thereby, detected a cryptic cytogenetic aberration involving chromosomes X and 7 effecting the novel occurrence of a chimeric fusion *MED14-HOXA9* in an adult patient.

## Materials and Methods

### Patient Characteristics

From March 2008 to November 2019, a total of 37 T/M MPAL patients were enrolled. Their median age was 45 years-old (range 17-84 years), comprising 22 males and 15 females. The study was approved by the Ethics Committee of the First Affiliated Hospital of Soochow University in accordance with the Declaration of Helsinki. Written informed consents were obtained from all patients.

### R-Banding Karyotype Analysis

Bone marrow (BM) samples were taken at diagnosis. Mononuclear cells were harvested, R-banded according to the routine institutional protocols and 20 metaphases were analyzed for each sample if possible. Chromosomal abnormalities were described according to the International System for Human Cytogenomic Nomenclature (ISCN, 2016) (7).

### RNA Sequencing and RT-PCR

Total RNA from BM samples taken at diagnosis was extracted using a RNeasy Mini Kit (QIAgen, Hilden, Germany). RNA sequencing libraries were prepared using 20-100 ng total RNA of BM samples with the TruSeq RNA library preparation kit v2 (Illumina, CA, USA). Paired-end sequencing with a read length of 150bp was performed on Illumina NovaSeq platforms to at least 12G raw data per sample according to the manufacturer’s protocol. Read pairs were aligned to human reference genome (hg38) using the Spliced Transcripts Alignment to a Reference (STAR, version 2.5) (8). The FusionCatcher software was used to find fusion genes. SNVs/indels were analyzed by following the GATK best practices for variant calling on RNA sequencing. The forward and reverse primer sequences used for detection of the *MED14-HOXA9* fusion by reverse transcription-polymerase chain reaction (RT-PCR) were 5’-AAGGTCTGTAAATGAGGACG-3’ and 5’-TCGTCTTTTGCTCGGTCTT-3’, respectively.

## Results

**Figure 1:**
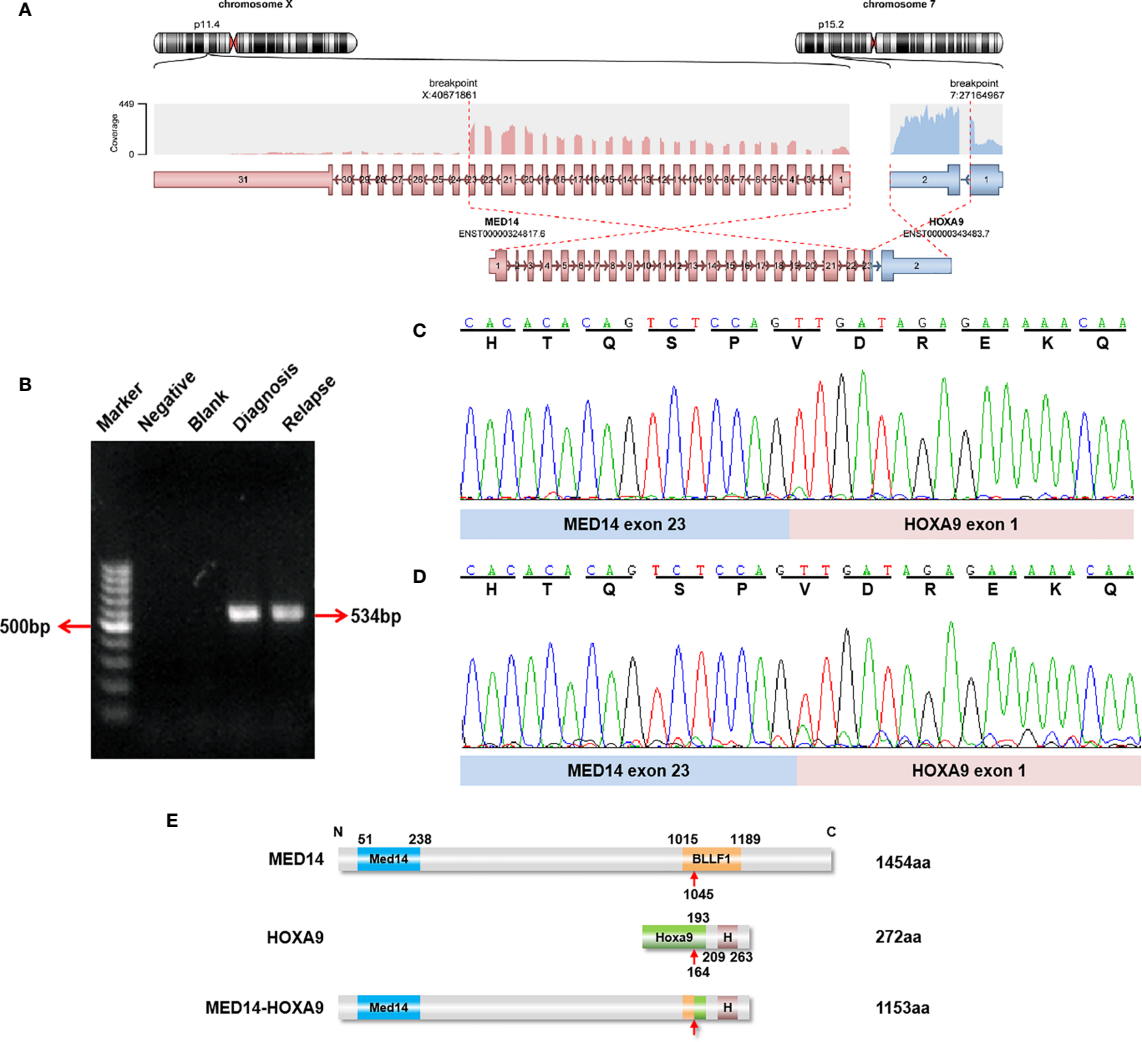
Identification of recurrent *MED14-HOXA9* fusion transcripts. **(A)** RNA sequencing analysis revealed one breakpoint in exon 23 of the *MED14* gene and one breakpoint in exon 1 of the *HOXA9* gene. **(B)** A product of 534 bp was detected by RT-PCR in samples taken at diagnosis and relapse. Marker: GeneRuler 100 bp DNA ladder; negative control (with cDNA sample with the JIH-5 cell line); blank control (without cDNA template). **(C, D)** Sequence alignment of the amplified product revealed breakpoints between exon 23 of *MED14* and exon 1 of *HOXA9* at diagnosis **(C)** and relapse **(D)**. **(E)** Schematic diagram of the *MED14* and *HOXA9* proteins and the *MED14*-*HOXA9* fusion protein. The breakpoint is indicated by a red arrow. Med14, Mediator complex subunit *MED14*; BLLF1, Herpes virus major outer envelope glycoprotein (BLLF1); HoxA9, HoxA9 activation region; H, Homeobox domain.

After R-banding, 17 patients (17/37, 45.9%) evidenced abnormal karyotypes, 17 tested normal (17/37, 45.9%), and 3 failed analysis. RNA sequencing was performed on the 20 patients with normal karyotypes or who failed karyotype analysis. In 19 patients, RNA sequencing failed to detect any clinically relevant fusion genes. In one patient with a normal karyotype, however, the *MED14-HOXA9* fusion transcript was detected using RNA sequencing: accordingly, exon 23 of *MED14* was fused in frame with exon 1 of *HOXA9* (**Figure 1A** and **Supplementary Table 1**), which was further confirmed with RT-PCR and Sanger sequencing (**Figures 1B, C**). Moreover, the *MED14-HOXA9* fusion was also detected in the blasts at relapse with RT-PCR (**Figure 1D**). The 1153 amino acids encoded by the *MED14-HOXA9* fusion transcript cover the entire Med14 and homeobox domains of *MED14* and *HOXA9*, respectively (**Figure 1E**). Though fusion genes are frequently resulted from balanced chromosome translocations, results of RNA sequencing and RT-PCR revealed no reciprocal *HOXA9-MED14* fusion transcripts in this patient (data not shown).

**Figure 2:**
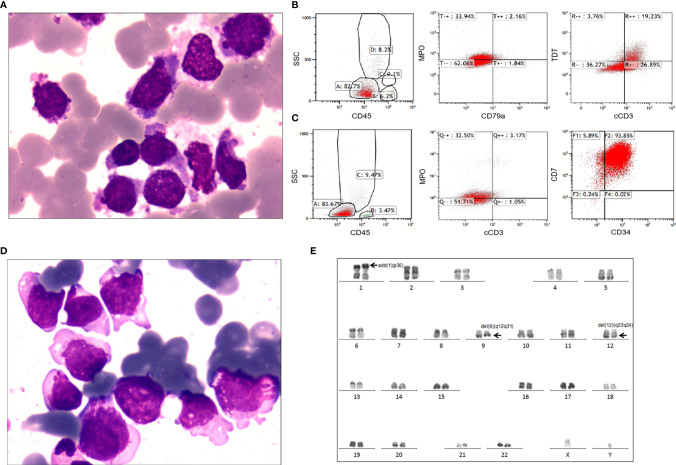
Laboratory characteristics of the adult patient with T/M MPAL with the *MED14*-*HOXA9* fusion. **(A)** Bone marrow aspirate at diagnosis. Morphological analysis showed hypercellular marrow with increased blasts variable in both cell and nucleolus sizes and negative for POX staining. (Wright’s staining ×1,000). **(B)** Immunophenotyping of bone marrow cells at diagnosis. Flow cytometric analyses of leukemic cells revealed that the blast cells were positive for TDT, cCD3 and weak positive for myeloperoxidase (MPO), but negative for cCD79a. **(C)** Immunophenotyping of bone marrow cells at relapse. The blast cells were positive for CD34 and CD7 and weakly positive for MPO and cCD3. **(D)** Bone marrow aspirate at relapse. Morphological analysis showed 72.5% blasts in the bone marrow smear. (Wright’s staining ×1,000). **(E)** Karyotype analysis at relapse. R-banding analysis using the bone marrow sample revealed a karyotype of 46,XY,add(1)(p36),del(9)(q12q31),del(12)(q23q24)[16]/46,idem,add(8)(q24)[2]/46,XY[2].

The affected patient was a 37-year-old male referred to our hospital in June 2016 because of mild fever. Physical examination revealed enlarged lymph nodes of the right neck, without hepatomegaly or splenomegaly. Complete blood cell counts showed a white blood cell count of 11.09×10^9^/L, hemoglobin of 10.9 g/dL and a platelet count of 84×10^9^/L (**Supplementary Table 2**). Differentiation analysis of white blood cells showed 59% blasts. BM aspirates revealed 71% blasts, which were variable in cell and nucleolus size and negative for peroxidase staining (**Figure 2A**). The blasts were positive for myeloid markers, including CD13 (97.8%), CD15 (68.9%), CD117 (63.2%) and weak expression of MPO (36.1%), as well as the T lineage markers cCD3 (46.1%) and TdT (23.0%) by flow cytometry (**Figure 2B** and **Supplementary Figure 1A**). No fusion genes were detected using a multiplex RT-PCR panel covering 43 fusion genes commonly seen in acute leukemia (**Supplementary Table 3**). Cytogenetic analysis of the BM indicated a normal karyotype. Next generation sequencing (NGS) was not feasible due to sample non-availability. *CREBBP-p.Pro583Ser, EED-p.Arg216Ter, NOTCH1-p.Val1578_Leu1579del* and *PTPN11-p.Ser506Leu* were detected by RNA sequencing (**Supplementary Table 4**). Accordingly, a diagnosis of T/M MPAL, NOS was made according to revised WHO 2016 criteria (2).

After induction chemotherapy, consisting of idarubicin, vinorelbine and prednisone, the patient achieved complete remission, which was maintained during the subsequent 5 cycles of consolidation chemotherapy. He refused allogenic stem cell transplantation due to concerns regarding transplantation-related mortality. Unfortunately, relapse occurred 11 months after diagnosis when he presented with high fever and general soreness. At relapse, the complete blood cell count showed a white blood cell count of 16.04×10^9^/L, a hemoglobin level of 12.5 g/dL, and a platelet count of 61×10^9^/L, with 26% blasts in the peripheral blood. Morphology analysis revealed 72.5% blasts in the BM (**Figure 2D**). Flow cytometry showed that the blasts were positive for CD7 (99.7%), CD13 (67.0%), CD33 (99.9%) and CD34 (93.9%), had weak expression of both MPO (35.7%) and cCD3 (4.2%) (**Figure 2C** and **Supplementary Figure 1B**), confirming the diagnosis of relapse. Cytogenetic analysis of the BM at relapse showed a karyotype of 46,XY,add(1)(p36),del(9)(q12q31),del (12)(q23q24)[16]/46,idem,add (8)(q24)[2]/46,XY[2] (**Figure 2E**). NGS of the relapse BM samples using a targeted 222-gene panel revealed mutations, including *CREBBP-p.Pro583Ser*, *EED-p.Arg216Ter*, *NOTCH1-p.Val1578Glu*, *PTPN11-p.Ser506Leu*, *ZNF292-p.Asp829fs* and *ZNF292-p.Met1243fs* (**Supplementary Tables 4**, **5**). The patient failed to respond to the initial induction chemotherapy regimen (idarubicin, vinorelbine, prednisone) combined with cyclophosphamide. He was also refractory to another chemotherapy regimen comprising homoharringtonine and cytarabine. The patient died of pulmonary infection at 34 days after relapse. Timeline is shown in **Supplementary Figure 2**.

## Discussion

T/M MPAL has both of myeloid and T lineage features immunophenotypically. Some studies applied NGS on the sorted blasts by flowcytometry to unravel the driver molecular lesions of T/M MPAL in the literature. The study of Xiao et al. showed that *PHF6* mutations were present in all blast compartments regardless of lineage differentiation, implicating that *PHF6* was an early mutation in T/M MPAL pathogenesis (9). Alexander et al. reported that biallelic *WT1* alterations were common in T/M MPAL (5). Because of the low incidence of T/M MPAL, these findings should be confirmed in larger scale studies using flowcytometry, NGS and single cell RNA sequencing. In this study, we performed RNA sequencing on diagnostic BM samples from 20 adult patients in order to explore the genomic landscape of T/M MPAL.

Here, we present the first case of *MED14-HOXA9* fusion to be reported in an adult T/M MPAL patient, confirming this fusion to be a rare but recurrent abnormality in T/M MPAL. *MED14* and *HOXA9* locate in chromosomes X and 7, respectively. Because there were no visible signs of abnormality in chromosomes X and 7 both at diagnosis and at relapse, we suggest that the *MED14-HOXA9* fusion resulted from a cryptic chromosomal rearrangement involving Xp11.4 and 7p15. *MED14-HOXA9* was detected in both the diagnostic and relapsed BM samples, therefore we believe that it might be one of the driver mutations responsible for leukemogenesis in this case. Because the patient presented with a normal karyotype at diagnosis but had acquired additional abnormalities at relapse, we reason that the structural aberrations involving chromosomes 1, 8, 9 and 12 observed at relapse were secondary to *MED14-HOXA9*, whether randomly occurring or contributory to disease progression.

*HOXA9*, located on chromosome 7p15, encodes a homeobox domain-containing and DNA-binding transcription factor, which plays an important role in regulating morphogenesis, differentiation and expansion of hematopoietic stem cells (10, 11). *HOXA9* is overexpressed in more than 50% of acute myeloid leukemia (AML) cases and is associated with poor prognoses (12, 13). A variety of upstream genetic alterations, such as *MLL* translocations, *NUP98* fusions, *NPM1* mutations and translocations involving *HOXA9* itself, have been demonstrated to cause overexpression of *HOXA9* (14). *HOXA9* reportedly forms translocations with 5 partner genes in myeloid malignancies: *ANGPT1*, *GATA2* and *NIPBL* in pediatric acute megakaryoblastic leukemia (15, 16); *NUP98* mostly in patients with AML and occasionally in patients with chronic myelogenous leukemia (CML) in blast crisis (17–19); and *MSI2* in a patient with CML in the accelerated phase (20). Other rearrangement partners of *HOXA9* have been reported in lymphoid malignancies, such as *TRB* in T cell acute lymphoblastic leukemia (T-ALL) patients (21) and *MED12* in a pediatric patient with B cell acute lymphoblastic leukemia (B-ALL) (22) (**Supplementary Table 6**). This report adds a new case, with a novel *HOXA9* rearrangement in T/M MPAL.

In previously reported *HOXA9*-related fusions, all *HOXA9* homeodomains were retained. *HOXA9-ANGPT1* apart, all other reported fusions comprise the N-terminus of the partner gene and exon 1 of *HOXA9* (located in the C-terminus). In this *MED14-HOXA9* case, the breakpoint also occurred in exon 1 of *HOXA9*, and the *HOXA9* segment was identical to that reported in other fusions, which indicates that the homeodomain of *HOXA9* is essential for leukemogenesis. *HOXA9* was shown to be upregulated by upstream fusions such as *NUP98-HOXA9*, which inhibits the differentiation of hematopoietic stem cells and increases the self-renewal of hematopoietic stem or progenitor cells (23). Collectively, these findings support the view that *HOXA9* is also aberrantly activated by the *MED14-HOXA9* fusion, which may play critical roles in the leukemogenesis of T/M MPAL.

MED14, mediator (MED) complex 14, plays an important role in coactivating RNA polymerase II (Pol II)-mediated transcription (24). In the *MED14-HOXA9* fusion gene, the main transcriptional domain of *MED14* was retained, which suggests an important role in *MED14*-mediated transactivation of *HOXA9* in this fusion. *MSI2*, another fusion partner of *HOXA9*, is also a putative RNA-binding protein. Thus, the *MED14-HOXA9* fusion adds another fusion gene in human leukemia encoding proteins with RNA-binding properties. A further MED-family gene, *MED12*, forms a fusion gene with *HOXA9* in B-ALL (22, 25). Mechanisms underlying the diversity of cellular settings with different *HOXA9* translocations might be attributable to the functions of the various translocation partners.

*MED14-HOXA9* fusion has been previously reported in a pediatric patient with T/M MPAL (5). Both the previously reported pediatric patient and the patient in this case were males diagnosed with T/M MPAL, had *PTPN11* mutations, relapsed with complex karyotypes, failed ALL chemotherapy and had short overall survivals (**Supplementary Table 7**). These shared characteristics indicate that patients with *MED14-HOXA9* fusion may: 1) be accompanied by *PTPN11* mutation serving as a critical alteration; and 2) follow an aggressive clinical course and confer an inferior outcome after conventional chemotherapy. Different *NOTCH1* mutations were detected in both diagnostic (p.Val1578_Leu1579del) and relapsed (p.Val1578Glu) BM samples, which both locate in the same hotspot in the heterodimerization domain of *NOTCH1* (**Supplementary Table 4** and **Supplementary Figure 3**). The different *NOTCH1* mutations at different disease timepoints suggest outgrowth of an alternate leukaemic clone carrying the missense mutation potentially due to some selective growth advantage or in response to therapy. Taken together, we speculated that in addition to the *MED14-HOXA9* fusion gene, mutations in *PTPN11* and *NOTCH1* co-operate and could be important for disease pathogenesis and potentially targeted treatments.

In summary, we report the first case of *MED14-HOXA9* in an adult T/M MPAL patient accompanied by precise clinical data, and confirm the presence of *MED14-HOXA9* fusion gene in both diagnostic and relapsed BM samples. Further studies will be carried out to reveal the mechanisms underlying the effects of the *MED14-HOXA9* fusion on the differentiation and proliferation of leukemia stem cells, as well as suitable treatment strategies for this rare disease.

## Data Availability Statement

The data generated by RNA-seq (raw FASTQ files) have been deposited to NCBI Sequence Read Archive (SRA) and have been assigned a BioProject accession number PRJNA726744.

## Ethics Statement

The studies involving human participants were reviewed and approved by the Ethics Committee of the First Affiliated Hospital of Soochow University. Written informed consent to participate in this study was provided by the participants’ legal guardian/next of kin. Written informed consent was obtained from the individual(s), and minor(s)’ legal guardian/next of kin, for the publication of any potentially identifiable images or data included in this article.

## Author Contributions

QW, LZ and M-qZ analyzed the clinical data and wrote the manuscript. ZZ and J-dX performed molecular studies and analyses. B-zF and NW performed the analysis of bone marrow smear and flow cytometry data. J-lP and C-xW performed karyotype analyses. RZ, S-nC and H-pD conceived and organized the work, and were major contributors in writing the manuscript. All authors contributed to the article and approved the submitted version.

## Funding

This study was supported by grant from the National Key R&D Program of China (2019YFA0111000), the National Natural Science Foundation of China (81000222, 81200370, 81700140, 81873449, 81970142, 81900130, 81970136, 82000132), the Natural Science Foundation of the Jiangsu Higher Education Institution of China (18KJA320005), the Natural Science Foundation of Jiangsu Province (BK20190180), China Postdoctoral Science Foundation (2018M632372), the Priority Academic Program Development of Jiangsu Higher Education Institution, Translational Research Grant of NCRCH (2020WSB11, 2020WSB13).

## Conflict of Interest

The authors declare that the research was conducted in the absence of any commercial or financial relationships that could be construed as a potential conflict of interest.

## Supplementary Material

Supplementary Figure 1 Immunophenotyping of bone marrow cells at diagnosis **(A)** and at relapse **(B)**, respectively.

Supplementary Figure 2 Timeline of the treatment regimens and responses. I, idarubicin; V, vinorelbine; P, prednisone; C, cyclophosphamide; H, homoharringtonine; A, cytarabine.

Supplementary Figure 3 Gene mutations and karyotype results in newly diagnostic and relapsed samples.

Supplementary Table 1 The result of RNA sequencing for MED14-HOXA9 fusion gene.

Supplementary Table 2 Clinical characteristics of the patient at diagnosis and at the time of relapse.

Supplementary Table 3 Detection of 43 fusion genes by a multiplex RT-PCR panel.

Supplementary Table 4 Gene mutations detected by NGS and RNA sequencing.

Supplementary Table 5 Detection of 222 genes by a NGS panel.

Supplementary Table 6 Reported cases of HOXA9 gene rearrangements in AML, ALL, CML, and MPAL.

Supplementary Table 7 Clinical characteristics of two patients with the MED14-HOXA9 fusion gene.
