# Supplementary figures and images for: A Recurrent Cryptic MED14-HOXA9 Rearrangement in an Adult Patient With Mixed-Phenotype Acute Leukemia, T/myeloid, NOS
Source: Front Oncol. 2021 Jul 22;11:690218. doi: 10.3389/fonc.2021.690218 (PMC8341862; doi:10.3389/fonc.2021.690218)

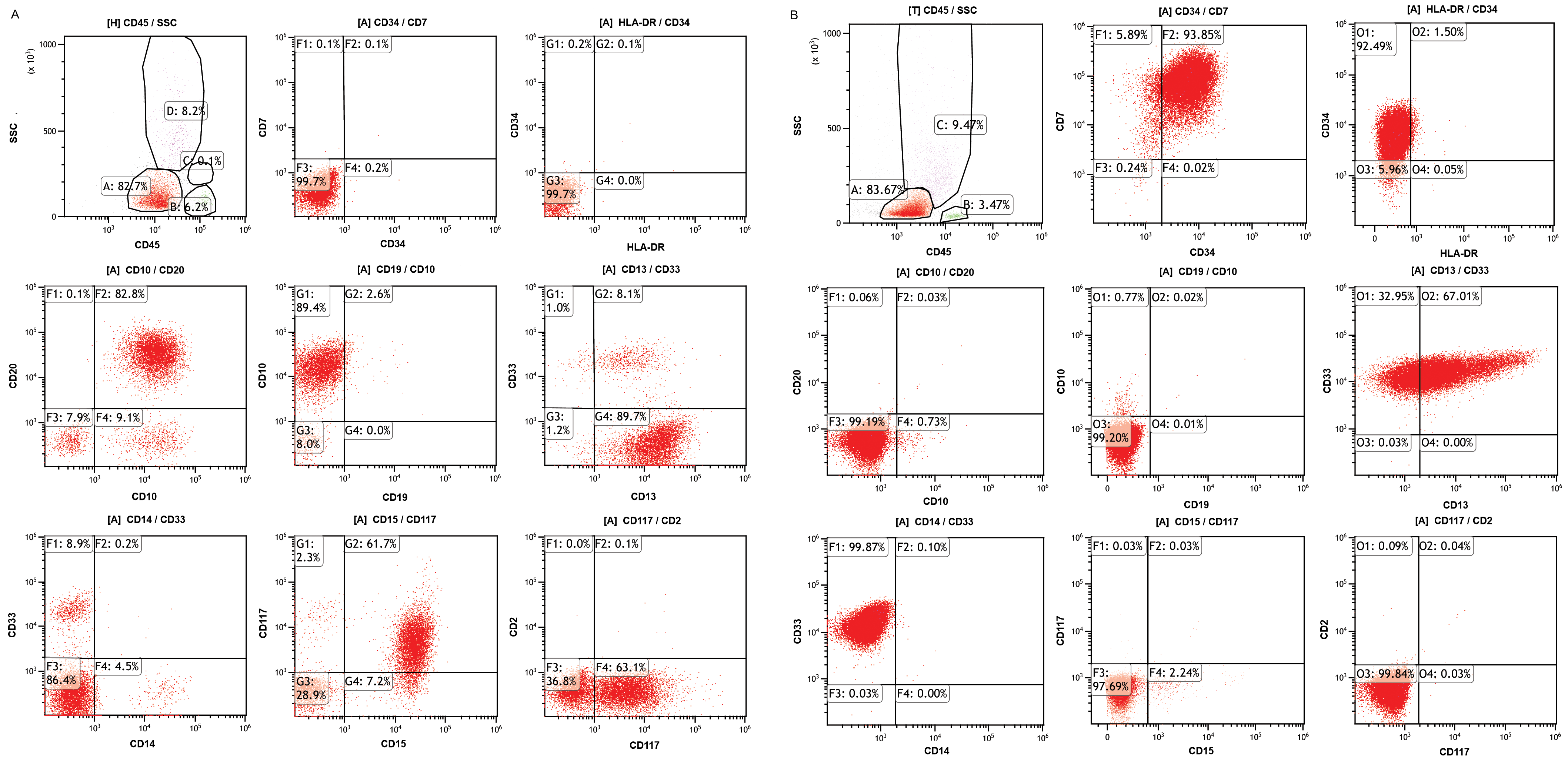

Supplement: Supplementary Figure 1 — Immunophenotyping of bone marrow cells at diagnosis (A) and at relapse (B), respectively. [file Image_1.tif]

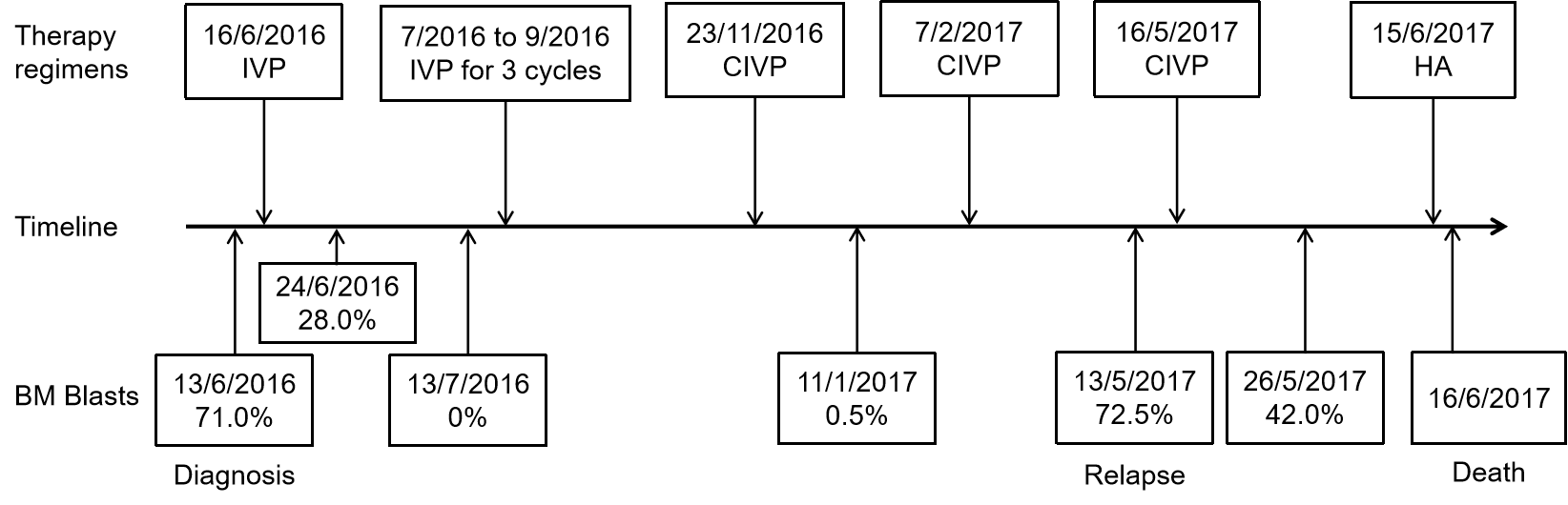

Supplement: Supplementary Figure 2 — Timeline of the treatment regimens and responses. I, idarubicin; V, vinorelbine; P, prednisone; C, cyclophosphamide; H, homoharringtonine; A, cytarabine. [file Image_2.png]

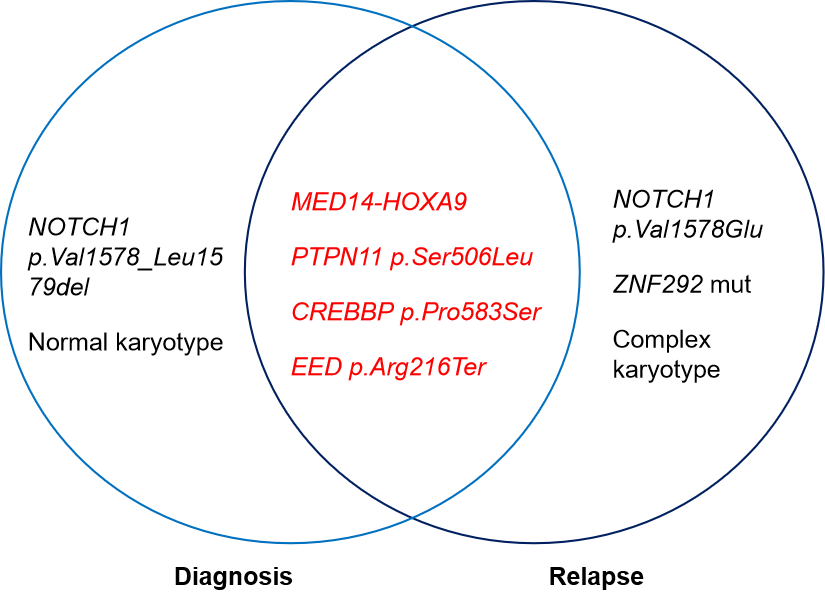

Supplement: Supplementary Figure 3 — Gene mutations and karyotype results in newly diagnostic and relapsed samples. [file Image_3.png]
